# Healthy eating interventions conducted in small restaurants and hot food takeaways: A systematic review

**DOI:** 10.1017/S1368980025000035

**Published:** 2025-01-09

**Authors:** Cinja Jostock, Hannah Forde, Nia Roberts, Susan A. Jebb, Rachel Pechey, Lauren Bandy

**Affiliations:** 1 Nuffield Department of Primary Care Health Sciences, University of Oxford, Oxford, UK; 2 Bodleian Health Care Libraries, University of Oxford, Oxford, UK

**Keywords:** healthy eating interventions, restaurants, hot food takeaways, systematic review, food environment, food purchasing

## Abstract

**Objective:**

This systematic review investigates the characteristics, effectiveness, and acceptability of interventions to encourage healthier eating in small, independent restaurants and takeaways.

**Design:**

We searched five databases (CENTRAL, Medline, Embase, CINAHL, and Science Citation Index & Social Science Citation Index) in June 2022. Eligible studies had to measure changes in sales, availability, nutritional quality, portion sizes, or dietary intake of interventions targeting customer behaviour or restaurant environments. We evaluated study quality using the Mixed Methods Appraisal Tool (MMAT). Results are synthesised narratively and interventions’ impact on personal autonomy is assessed using the Nuffield intervention ladder.

**Setting:**

Small, independent or local restaurants or hot food takeaway outlets, with no restrictions by year or country.

**Participants:**

Anyone selling or purchasing food in intervention settings (e.g. restaurant staff/owners, customers).

**Results:**

We screened 4,624 records and included 12 studies describing 13 interventions in 351 businesses. Most studies were of poor quality. Customer-level intervention components mostly operated on the lower rungs of the Nuffield ladder and most had limited positive effects on increasing demand, measured as sales or orders of healthy options. Whilst rare, most interventions measuring business outcomes operated on higher ladder rungs and showed small positive results. There was insufficient evidence to investigate differences in impact by intervention intrusiveness. Acceptability was greater for interventions that were low-effort, inexpensive, and perceived as not negatively impacting on customer satisfaction.

**Conclusions:**

Despite some evidence of small positive effects of healthy eating interventions on healthier purchases or restaurant/hot food takeaway practices, a weak evidence base hinders robust inference.

## Background

One in five deaths worldwide are linked to poor diet ^(1)^. Food consumed out of the home in restaurants, cafes, and takeaways tends to be high in calories ^(2)^, saturated fat ^(3)^, and salt ^(4)^, with more regular consumption linked to increased risk of higher body weight ^(5)^. A 2022 survey conducted by the Food Standards Agency in England, Wales, and Northern Ireland found that 53% of respondents had eaten in a restaurant, 50% had ordered takeaway food, and 38% had consumed food from a fast-food outlet in the previous four weeks ^(6)^. Evidence from high-income countries also suggests fast-food outlets are more common in deprived communities compared to more affluent areas ^(7–9)^.

Small businesses dominate the sector, with just over half of the £62 billion of revenue generated from the UK consumer foodservice sector in 2022 coming from small and locally owned restaurants and hot food takeaways ^(10)^. Unlike large chain restaurants, small independent businesses usually operate in small premises, with limited staff, equipment, and access to suppliers ^(11)^, and finite resources to participate in healthy eating interventions specifically ^(12)^. Yet in contrast to large, chained businesses, owners of small independent businesses may be more likely to be able to make decisions about whether and how to enact interventions ^(13)^.

A study found that independent and small-chain restaurants (under 20 outlets) serve meals with higher energy content than those in larger chain restaurants, with individual meals accounting for 66% of an adult’s daily energy requirements ^(14)^. Nevertheless, existing reviews on healthier eating interventions in restaurants, takeaways and fast-food outlets mostly rely on large chain restaurants ^(15)^ or include both chains and non-chains ^(16, 17)^, and policies aiming to support healthier food purchasing in the out of home sector (e.g. nutrition labelling) have typically only applied to larger businesses who have greater resources to implement such legislation ^(18, 19)^. However, this risks widening health inequalities if the small businesses exempt from them provide less healthy food and are more abundant in predominantly poorer areas. Indeed, evidence from Australia shows that independent takeaways are more common in deprived than affluent areas ^(20)^ and studies from the UK describe a high prevalence of independent fast-food and takeaway outlets in disadvantaged areas ^(21, 22)^. Therefore, separate interventions are needed for small, independent restaurants, which are effective in improving food healthiness whilst being feasible and acceptable to restaurants. For example, in the case of menu labelling, a survey among independent restaurants in Canada found that common worries are the expenses and time effort required to implement such a policy ^(23)^.

The differences between chained and independent restaurants and takeaways mean that policymakers need specific information on the types of interventions that may be effective in small restaurants and hot food takeaways, including potential challenges and opportunities to developing effective interventions. We aimed to systematically review the extant evidence of interventions to promote healthier food purchasing or consumption in this setting.

The objectives of this review were to:

1) Establish the characteristics of healthy eating interventions conducted in small, independent, or local restaurants and hot food takeaways (hereafter ‘restaurants and takeaways’)
2) Assess the impact these interventions had on food availability and purchasing patterns
3) Identify characteristics of interventions that increased acceptability to small restaurant and takeaway staff and owners

The findings of this review can inform policymakers on which interventions may be effective and acceptable in small independent restaurants and takeaways and can be implemented at a local level.

## Methods

### Protocol and registration

The pre-registered protocol is available on PROSPERO [CRD42022341791]. This review follows the Preferred Reporting Items for Systematic reviews and Meta-Analyses (PRISMA) 2020 checklist for reporting of systematic reviews ^(24)^ (Appendix 1).

A review with a wider scope was specified in the protocol including healthier eating interventions in small food stores and restaurants. However, after completing data extraction, we split the review into two papers to focus on each setting in isolation. Instead of using the National Institutes of Health (NIH) Quality Assessment Tools ^(25)^ as pre-specified, we used the Mixed Methods Appraisal Tool (MMAT) ^(26)^, which enabled us to evaluate different study types employing one tool and guidance document.

### Data sources and search strategy

The search strategy for this review was tailored to identify interventions in small restaurants and takeaways, and food stores. An information specialist (NR) developed the search strategy in consultation with LB after initial scoping searches. We searched the following databases for primary studies from database inception to 15th June 2022: Cochrane Central Register of Controlled Trials (CENTRAL), Issue 5, 2022, the Cochrane Library, Wiley, MEDLINE and Epub Ahead of Print, In-Process & Other Non-Indexed Citations and Daily, OvidSP (1946-), Embase, OvidSP (1974-), CINAHL, EBSCOHost (1982-) and Science Citation Index & Social Science Citation Index, Web of Science Core Collection (1900-). Search strategies were comprised of keywords and controlled vocabulary terms. We applied no limits on language or publication date. We used the filter designed by the Cochrane EPOC group to identify randomised studies, before and after studies and interrupted time series (https://zenodo.org/record/5106292). We used the Polyglot tool from SR-accelerator (https://sr-accelerator.com/#/polyglot) to adapt the search formatting from Medline to the other databases. All search strategies are provided in Appendix 2A.

All references were downloaded to Endnote 20 ^(27)^ before being transferred to Covidence ^(28)^. In November 2022, we additionally conducted forward and backward searches of eligible studies and six reviews ^(15, 16, 29–32)^ using Citation Chaser ^(33)^, with results subsequently imported into Covidence ^(28)^. We excluded two more restaurant and takeaway papers ^(34, 35)^ after the citation tracking due to them not meeting our setting or outcome requirements.

### Eligibility criteria

Eligibility criteria were determined following the PICOS framework ^(36)^ and are outlined in Table 1 below. We included primary studies where the study authors described the restaurant or takeaway as small, independent, community-based or local and where there was no evidence that the restaurants or takeaways were part of a chain. A community-based restaurant was defined as a place for local people to come together to eat freshly cooked food.

### Study selection and data extraction

After exclusion of duplicates, abstract and full text screening was completed independently and in duplicate by two reviewers (LB and CJ) using Covidence ^(28)^. Any conflicts were discussed by the two reviewers, and a third reviewer arbitrated if needed.

Data extraction was conducted by one reviewer, with reviewers subsequently checking the data extraction forms completed by the other reviewer. Again, any conflicts were discussed and resolved between the two reviewers. Data extracted included first author name and year, country and location, stakeholders involved, study aim, methods (design, start and end date, targeted population, co-design and stakeholder involvement, if applicable), setting type, sample size, recruitment methods, inclusion criteria, intervention characteristics (name, description, duration, comparator/control), outcomes, measured data, statistical or analysis methods, main findings, barriers and facilitators to working with businesses, and recommendations for future studies.

### Study quality assessment

Study quality was rated independently by one reviewer and verified by a second reviewer (LB or CJ) using the MMAT ^(26)^. Each of the included studies was first categorised into one of five groups based on study design: 1) qualitative research; 2) randomised controlled trials (RCTs); 3) non-randomised studies; 4) quantitative descriptive studies; and 5) mixed methods studies. Studies were then assessed for quality using the category-specific criteria and presented in full, as is recommended, rather than being adapted into a single score ^(26)^.

### Synthesis of results

Results were synthesised narratively ^(37)^. Main characteristics and outcomes of interventions were summarised in tables and patterns identified. Additionally, our analysis was guided by the Nuffield intervention ladder ^(38)^, which categorises interventions according to their intrusiveness (i.e. their impact on individual freedom) (Table 2). Briefly, higher steps on the ladder represent more intrusive interventions, with eliminating choice being the highest step (i.e. most intrusive intervention) ^(38)^. Each element of the included interventions was grouped depending on whether it was designed to impact consumer behaviour (customer-level interventions) or the business’s behaviour (business-level intervention).

## Results

**Figure 1:**
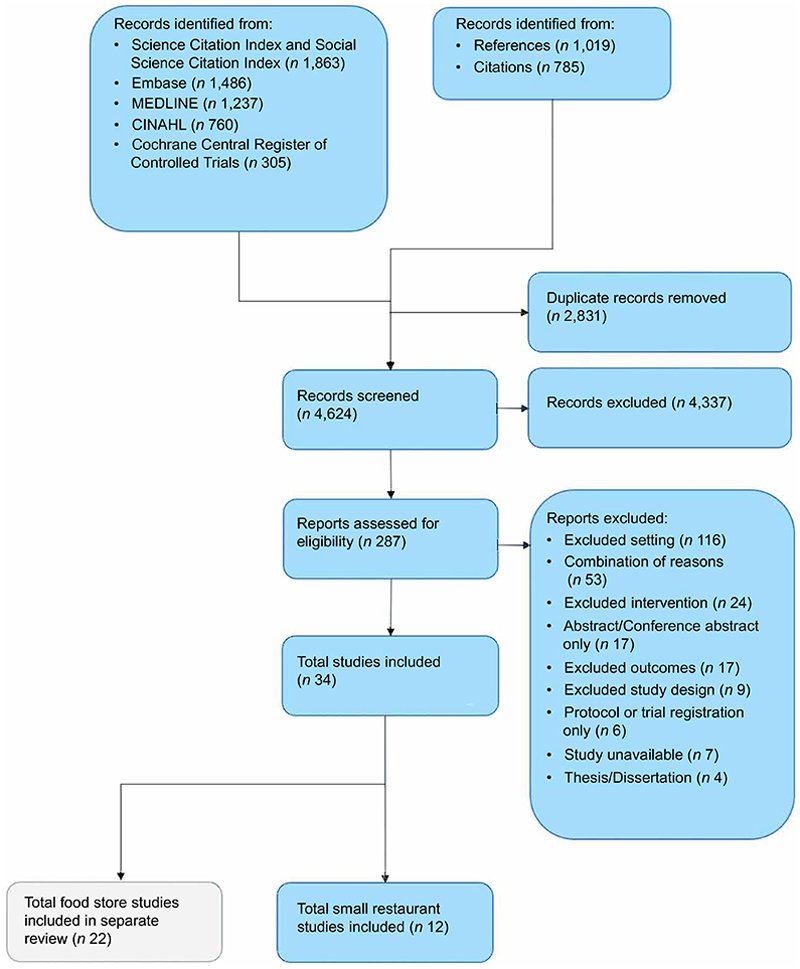
PRISMA flow diagram showing the study selection process ^(24)^

Searches retrieved a total of 7,455 records, and after removing duplicates, 4,624 records were screened (Figure 1). We assessed 287 full-text records for eligibility, resulting in the inclusion of 12 studies reporting on 13 interventions.

### Quality assessment

Of the 12 studies included, most used a mixed-methods ^(39–43)^ (n=5) or a quantitative, non-randomised ^(13, 44–46)^ (n=4) study design (Table 3). Two were RCTs ^(47, 48)^ and one study was quantitative descriptive ^(49)^. No qualitative studies were identified, potentially due to our outcomes of interest being geared towards quantitative measurements.

Neither of the RCTs ^(47, 48)^ met all of the MMAT’s ^(26)^ quality criteria. Randomisation was either not appropriately performed ^(47)^ or insufficiently described ^(48)^, and neither study reported whether outcome assessors were blinded to the intervention, which limited their quality assessment scores.

Only one of the four quantitative, non-randomised studies provided sufficient detail to be appraised and met all five criteria ^(44)^. Other studies provided insufficient detail on the population’s representativeness ^(46)^, whether there was complete outcome data ^(13, 45)^, whether confounders had been accounted for ^(13, 46)^, and whether the intervention was implemented as intended ^(46)^.

One study was a quantitative descriptive study ^(49)^. It met the criteria on sampling strategy and statistical analysis, but provided insufficient information or did not meet the criteria for representativeness of the sample, appropriateness of variables and measurements, and the risk of non-response bias.

Five studies were mixed methods ^(39–43)^. While all were strong on integrating qualitative and quantitative components of their research questions, they did not provide sufficient information on or failed to address the divergences and inconsistencies between quantitative and qualitative findings. Some also failed to meet the criteria on providing an adequate rationale for using mixed methods ^(39, 40, 43)^, integrating quantitative and qualitative interpretation ^(41)^, and adhering to the quality criteria of the different methods involved ^(39, 40, 43)^.

### Settings and stakeholders involved in the interventions

Twelve studies reporting on 13 interventions were included, with two papers assessing the same intervention ^(44, 45)^ and two papers testing two interventions each ^(47, 48)^. Eight interventions involved small restaurants ^(40, 41, 46–49)^, three focused on takeaway outlets ^(13, 39, 44, 45)^, and two included both ^(42, 43)^. The number of businesses involved varied, ranging from one ^(48, 49)^ to 206 ^(13)^ (Table 4).

Ten interventions were conducted in the USA ^(13, 40, 41, 44–49)^ and three in the UK ^(39, 42, 43)^. Most took place in cities ^(13, 41, 44, 45, 49)^, highly populated counties ^(47, 48)^, boroughs ^(42)^, or suburban areas ^(46)^. One intervention was set in rural small-town settings ^(40)^, and one included both urban and rural settings ^(43)^. Although not all studies provided this information, several targeted low-income areas ^(13, 41, 44, 45)^, and others spanned areas with various levels of deprivation ^(39, 42, 43)^. All but three interventions ^(40, 47)^ engaged a wider range of stakeholders other than businesses and academic researchers, commonly from the local authority (n=6; e.g. health teams, environmental health officers) ^(13, 39, 41, 42, 46, 49)^ or local community organisations or NGOs (n=3) ^(13, 41, 49)^.

Some interventions focused on specific cuisines, such as American ^(40, 47)^, Latino ^(47)^, Chinese ^(13)^, or British ‘Fish & Chip’ shops ^(43)^. Two interventions had inclusion criteria relating to business owner ethnicity, targeting African-American or Korean-American takeaway owners ^(44, 45)^ or Chinese American restaurant owners or chefs ^(13)^. Several interventions conducted in the US were set in areas with a high or growing proportion of residents identifying as Latino or Hispanic ^(41, 47–49)^, African American ^(44, 45)^, or areas with a high proportion of ethnic minority residents ^(13)^.

### Interventions based on their classification on the Nuffield intervention ladder

Almost all interventions had components classed as ‘customer-focused’ as well as ‘business-focused’ ^(13, 39, 41–49)^, with one intervention solely aimed at the customer-level ^(40)^ (Table 5). All but three interventions operated on more than one rung of the Nuffield ladder ^(40, 47, 48)^. The highest rung used was restricting choice on the business-level ^(39, 42)^. The lower Nuffield ladder classifications which ‘provide information’ and ‘enable choice’ were most commonly used, aimed at both customers (e.g. menu labelling) and business owners and staff (e.g. cooking guidelines for chefs) (Table 5).

### Business-level intervention components & outcome measures

Five interventions measured business-level outcomes ^(13, 39, 41–43)^ (Table 6). Three studies used the number of businesses meeting certain criteria as an outcome measure ^(39, 42, 43)^ and three studies measured the nutrient content or weight of dishes sold ^(13, 41, 43)^, with one also describing self-reported changes to cooking habits ^(41)^. Four studies only provided descriptive evaluations ^(39, 41–43)^.

Four interventions resulted in small increases in the number of businesses complying with criteria ^(39, 42, 43)^, reduced weight of sold meals ^(43)^, or sodium content of dishes ^(13)^. One intervention described staff reporting positive changes to cooking habits ^(41)^.

#### Restrict choice

Three interventions ^(13, 39, 42)^ aimed to reduce the sugar, fat, and salt content of foods, for example by changing cooking practices (e.g. cooking oil usage) or switching to healthier products. Two used the number of businesses and number of criteria met as outcome measures and reported small positive effects ^(39, 42)^, whilst another recorded lower sodium content of dishes ^(13)^. However, only one conducted statistical testing ^(13)^.

The *Healthy Catering Commitment (HCC)* in London is a series of criteria relating to cooking, serving, and selling practices; businesses are expected to meet eight out of 22 criteria before being awarded a Healthy Catering (HCC) Award by their local authority ^(42)^. 77 businesses were surveyed, each having to make an average of 2.5-criteria related changes to secure the award ^(42)^. More businesses (n=26) signed up to ‘provision of information’ (e.g. promotion of healthy eating by staff) compared to ‘enabling choice’ criteria (n=1-15, depending on change) (e.g. offering fresh fruit, smaller portion sizes) due to cost and potentially reduced revenue associated with the latter. Criteria to ‘eliminate choice’ that were cheap and perceived as not interfering with customer preferences (e.g. cooking oil practices) were readily implemented; however there was more hesitancy for changes visible to customers (e.g. thick-cut chips).

Similarly, the *Takeaway Masterclass intervention* asked businesses to commit to health-promoting practices and provided interactive training ^(39)^. Businesses committed to a median of 4 goals/criteria (range 1 to 7) and achieved a median of 3 goals, including increasing vegetables in meals and grilling and poaching instead of frying ^(39)^.

The *Healthy Chinese Take-Out Initiative* included a media campaign and low-sodium training, with takeaways adopting sodium-reduction techniques such as lowering the amount of soy sauce used ^(13)^. A significant and sustained reduction in the sodium content of three target dishes was found, with relative reductions of 36% for Dish 1 (5.5 to 3.5mg/g), 28% for Dish 2 (5.7 to 4.1mg/g) and 19% for Dish 3 (5.9 to 4.8mg/g), although all three dishes remained above the local authority’s recommended sodium intake per meal ^(13)^.

#### Guide choice through incentives

The *Fish and Chip Wholesaler Study* combined public pledging, provision of smaller-sized packaging, and an information and engagement session ^(43)^. Although only reporting descriptive statistics, the number of venues offering smaller portion meals increased from 6 at baseline to 8 at 6-week post-intervention and the weight of fish and chip meals sold decreased a mean of 37g for regular meals and 27g for small meals ^(43)^.

#### Enable choice

*Salud Tiene Sabor*, a menu labelling intervention, reported restaurant staff declaring they employ healthier cooking practices as a result of the intervention, including using more vegetables ^(41)^. They also tested meals served for their calorie content and found that post-intervention, 58% of main meals and 59% of side dishes remained above the local authority’s recommended calorie content per meal, although there was no baseline or control for comparison ^(41)^.

### Customer-related intervention components & outcomes

Ten interventions measured customer-related outcomes ^(40, 41, 43–49)^ (Table 6). Nine interventions used value sales and/or order data to measure intervention impact on customer-related behaviour change ^(40, 43–49)^, and six reported customer-interview data ^(40, 41, 43, 45, 48)^. Studies that used sales data reported challenges with data collection. There was heterogeneity in registers/tills across restaurants that made the data hard to process ^(47)^, not all intervention restaurants and takeaways provided data ^(43)^, and it was laborious to manually input paper order slips ^(44)^.

One intervention reported a positive effect on smaller portion size orders ^(43)^, although only evaluated descriptively. Three interventions found mixed results (positive and no changes) on healthy foods sold ^(44, 48)^. Two interventions recorded no significant increases in healthy item orders ^(40, 46)^. Three interventions reported orders occurred from the new healthier menu but it was unclear if these replaced or supplemented orders from the existing menu ^(47, 49)^. One intervention reported that nutrition information influenced the purchase decisions of one-third of customers, but there was no baseline comparison ^(41)^.

#### Guide choice through incentives

Two interventions provided financial incentives for healthy meal choices through price promotions ^(44, 45)^ and donations to local causes ^(48)^, finding mixed effects (positive and no changes) on sales of healthier items depending on item targeted ^(44)^ or comparison time period ^(48)^.

The *Baltimore Healthy Carryout* intervention recorded a statistically significant interaction between groups for healthier sides and beverages sold in two of three intervention phases, but not for healthier entrees or healthy items overall ^(44)^. The greatest increase was seen in phase three, where a price promotion (incentive) was implemented alongside healthier cooking methods ^(44, 45)^. Although the effect of intervention phases cannot be isolated due to each new phase building on the previous one, this could suggest that intervention elements higher up the ladder may have been more successful within this study. In the process evaluation, 42.6% and 65.3% of surveyed customers reported choosing an option due to the BHC leaf logo or photos on the menu respectively ^(45)^. The *Fundraising Healthy Eating Scheme* intervention made higher financial donations to local schools contingent on orders of healthier menu items, and found a higher percentage of healthier menu items were ordered during the intervention than in the post-intervention period but not in the pre-intervention period ^(48)^. Six of the surveyed customers (20.7%) said they chose their meal option due to the incentive ^(48)^. There was no significant difference in orders of healthy items between the intervention arms (other arm reported under *provide information*) ^(48)^; suggesting adding a higher ladder level component (incentive) did not provide additional benefit in this instance.

#### Enable choice

Five interventions enabled choice by adding new healthier meals or sides to menus ^(47, 49)^ or marking healthy options on menus ^(41, 44–47)^. Three recorded orders of new healthy items, but it remained unclear how order numbers from existing menus were affected ^(47, 49)^. One intervention found no changes in healthy item orders ^(46)^. In one study, a third of clients stated their order was influenced by nutrition information, but there was no comparison ^(41)^.

The *Kids Choice Restaurant Program (KCRP)* created new healthier menus in both interventions, with one intervention additionally employing marketing and employee training ^(47)^. Both interventions recorded increased sales of new healthier menu items immediately after implementation, with the proportion of healthier items making up 23% (menu plus) and 17% (menu only) of all child’s menu items in the first four intervention weeks ^(47)^. However, sales of pre-existing menu items did not differ between the two conditions during the intervention, and difference from baseline was not assessed statistically ^(47)^. The *Galerias Restaurant intervention* found that after 6 weeks, 11.6% of item orders were from the new intervention menu, but there was no comparator and it is unclear whether sales of less healthy items decreased ^(49)^. The *Healthy Dining Program* labelled and promoted healthy menu items and found no change in targeted healthy menu orders pre-intervention to six-weeks post-intervention ^(46)^. *Salud Tiene Sabor* found that one third of customers stated their purchases were influenced by the point-of-sale nutrition information that was displayed during the intervention, but there was no pre-intervention comparator ^(41)^.

#### Provide information

Three interventions provided information only, promoting healthier products using marketing materials such as table tents ^(40, 43, 48)^ or providing point-of-purchase nutrition information ^(48)^. One intervention described slightly increased small portion orders ^(43)^, however not using any statistical tests. One intervention found mixed results (positive and no effect) ^(48)^ and one no effects on healthier orders ^(40)^.

The *Fish and Chip Wholesaler Study* encouraging fish & chip shop owners to offer and promote smaller portion sizes found increases in the number of small-portion meal orders from 14.2% of total Fish & Chip orders before the intervention to 21.2% post-intervention, with 20% of surveyed customers indicating they had tried a smaller portion meal ^(43)^. The *Signposting to Healthy Meals* did not have a comparator group and found no significant changes in order slips, although 34% of customers who were aware of the signs said that these had impacted their order ^(40)^. One intervention arm from the *Fundraising Healthy Eating Scheme* provided information on healthier items and a 15% donation of the total bill value to the school wellness programme, and recorded significantly increased healthy item orders compared to follow-up but not the baseline period ^(48)^. Only four surveyed customers (10.8%) said they selected their option due to the promotion materials ^(48)^.

### Intervention barriers and facilitators

Recruitment of restaurants and takeaways can be challenging, with recruitment rates for businesses varying from 10% ^(39)^ to 100% ^(40)^ of those approached to participate in the evaluation. Four studies did not report recruitment rates ^(13, 41, 46, 48)^. One research team was approached by a business owner wanting to conduct an intervention ^(49)^. Identifying and visiting potential restaurants and takeaways several times before recruitment was reported as a strategy for successful recruitment ^(44, 45)^. One other study reported that a local restaurant association played a strategic role in recruiting restaurants ^(13)^.

Five studies reported intervention fidelity, all achieving moderate to high fidelity ^(40, 43, 45, 47, 48)^. Three studies reported barriers relating to difficulties engaging busy restaurant and takeaway staff with the training ^(47)^, high turnover rates ^(13)^ and trusting that staff would correctly deliver smaller portion sizes as intended ^(43)^. Framing the intervention as ‘good customer service’ was reported to be potentially beneficial to serving staff implementing the intervention as intended ^(43)^. Motivated staff, especially owners and managers, were key to keeping businesses engaged with and implementing the intervention ^(39, 45, 48, 49)^. Building good relationships with owners and involving them in decisions ^(45)^ as well as building strong partnerships ^(13)^, with for example support from community groups ^(41, 49)^ or working with a wholesaler ^(43)^, were also mentioned as facilitators.

Two studies reported that businesses better engaged with intervention elements that were cheap, easy to implement, and perceived as acceptable or less noticeable to clients, which included easy-to-implement intrusive interventions (e.g. changing cooking oil used, categorised as *restrict choice*) ^(39, 42)^. One study reported that a two-phase intervention where low-cost, low-burden intervention elements are implemented first while building a stronger rapport with business owners and managers before introducing higher-burden intervention elements was effective at keeping businesses engaged ^(44, 45)^. Conversely, worries about customer satisfaction, a lack of demand for healthier products and associated costs were common barriers ^(39–42, 45)^. Six studies reported that an intervention’s economic impact is an important factor for business owners when considering whether to engage with interventions, primarily because of small restaurants’ and takeaways’ small profit margins and susceptibility to economic fluctuation ^(40–45, 49)^. Businesses were reported to be motivated by the potential (financial) benefit of an intervention ^(43, 48)^, positive feedback from clients ^(39)^, and financial incentives (e.g. supplies, covering first stock) ^(45)^.

One main barrier to implementation was a lack of availability of healthier products from suppliers, either at all, or at a comparative price point to regular versions ^(39, 42)^. This may be more common in more rural areas and outside of large cities ^(39)^ and both businesses and customers in more affluent areas may be more willing to pay the extra costs involved in healthier options ^(42)^.

A web-based tool kit was a useful tool for dissemination of lessons learned and for potential participating businesses to learn more about the intervention ^(41)^.

## Discussion

### Summary of findings

Interventions to encourage healthy eating in small, independent restaurants and takeaways were mostly a complex mix of initiatives integrating business-level elements and consumer-focused components. Study quality was poor with limited quantitative outcome data and it was not possible to conduct a meta-regression to identify effective components. Nonetheless, we found some narrative themes. Interventions focused at the customer-level were mostly at the lower rungs of the Nuffield ladder. Enabling choice through introducing new and healthier menu items resulted in healthier items being ordered, with take-up varying from 11.6% ^(49)^ to 23% ^(47)^ of orders, but it was less clear whether these items substituted or supplemented other less healthy items. There was also a lack of evidence on whether the uplift in sales when new menu items were introduced could be sustained. Providing incentives (at the mid-point of the ladder) also resulted in a mix of positive results and no effect, with impact varying across product categories or comparison periods. Price promotions appeared to have some effect at least in the short-term to boost sales of healthy products ^(44)^, but may not be a sustainable option for small businesses with tight margins. Most business-level interventions were classified as operating at mid-to-high rungs of the Nuffield ladder. Few interventions evaluated business-level outcomes but almost all reported some positive effect including greater adherence to nutritional criteria, or reduced salt content or weight of dishes, though quantitative evidence of effectiveness was scarce.

### Strengths and limitations

We comprehensively searched relevant academic databases, including through multiple screeners and new software (e.g., Citation Chaser ^(33)^), building confidence in the scope and accuracy of our review. Our synthesis of studies provides the first overview to identify characteristics that are important for successful intervention design and implementation to improve food healthiness in small restaurants. We used the Nuffield intervention ladder to categorise intervention components; two included studies similarly used the Nuffield ladder to characterise intervention components ^(39, 42)^ whilst another study reflected on their results using the Nuffield ladder ^(43)^, highlighting the relevance of this framework. Although we risk excluding studies by not conducting additional grey literature searches, higher-quality studies would likely be published in peer-reviewed journals.

The small number of heterogeneous and relatively low quality studies identified in the review is in itself a finding of interest, but limits the potential generalisability of these results. Few studies had a randomised design, and it was not possible to directly compare interventions due to the heterogeneity of intervention components, study designs, and settings. Furthermore, the narrow geographic range (urban areas in UK and US) of studies included means findings may not translate to other food cultures (e.g., informal food economies). Additionally, our review may be limited by publication bias ^(50)^, particularly considering most interventions described at least some positive effects.

### Interpretation and comparison to existing literature

To the best of our knowledge, this is the first review that has focussed specifically on small, independent restaurants and takeaways. The poor quality of available evidence and lack of impact evaluations in the out of home field has been reported in previous reviews ^(15–17, 51)^. For example, in a review summarising interventions in food outlets in England, only 21 out of 75 interventions included evaluations of the impact or outcome of the intervention and such evaluations were done to aid service delivery rather than research-led initiatives ^(51)^. Challenges with data collection as reported by many studies in this review may impede rigorous evaluation.

Previous reviews also found that “simple” environmental changes such as information provision and promoting existing healthy options are particularly common intervention strategies in community-based restaurants ^(16, 17)^, consistent with our finding that easily implemented and cheap interventions are most acceptable to businesses. One reason the provision of information appeared to have mixed effects across studies in our review may be that customers often arrive at food outlets with pre-established order intentions; therefore, material to highlight new menu options and point of sale nutrition information may have a limited effect ^(47)^. Additionally, nutrition labels may be ignored if the main eating motivation is hedonic and quick decisions are required ^(52)^. Indeed, research shows that taste is valued more strongly than health for restaurant meals ^(53)^. Therefore, intervening to encourage healthier eating may be particularly challenging in these settings.

The studies that reported intervention fidelity found compliance to be moderate to high. However, engagement varied both between intervention venues and different intervention components, highlighting the need for a tailored approach. The relatively flexible format of some interventions - for example where restaurants were given some liberty to choose which changes they would like to implementor adapt -, meant that restaurants were able to select changes that best fit their context. Studies also reported that it is easier to engage participating businesses with interventions that are low-cost, low-effort, and unlikely to be rejected or noticed by customers ^(39, 42)^, and one intervention reported success using a staggered approach that slowly introduced more intrusive components ^(44, 45)^. Whilst rare, some higher-level interventions were identified, demonstrating these can be implemented. However, higher-level interventions requiring structural changes may be beyond the financial resources of small restaurants and takeaways. One strategy could be creating greater and equal opportunity for small restaurants and takeaways to access and serve healthier foods, which are often more expensive or only come in large package sizes unsuitable (and unaffordable) for small businesses ^(11)^, for example through the provision of wholesaler subsidies for healthy foods for small restaurants and takeaways. An intervention providing discounts on healthy foods for small stores at wholesalers found this led to increased availability of healthier options ^(54)^.

Economic incentives or perceived economic viability of interventions was a main facilitator for engagement. Additionally, establishing rapport with owners may benefit recruitment ^(44, 45)^, a finding corroborated by previous evidence stressing the need for community outreach ^(11)^. Although studies reported which stakeholders were involved in the intervention in their backgrounds and methods, there was a lack of discussion and identification of the roles and benefits that other stakeholders played. Greater information about motivations and barriers to stakeholder involvement could improve the design and delivery of interventions in future.

### Implications for policy and research

Most studies relied on descriptive statistics, short follow-up periods, and had no control or comparator sites, likely partly due to resource constraints and recruitment difficulties. More high-quality studies of interventions are needed, evaluating the longer-term impacts and sustainability of interventions using objective measures of outcomes (e.g. sales data). Investing in a new data system, or training staff on how to input data so that it is usable for the study, is advised if possible within resources available – improved sales data may also help inform businesses’ strategies, as well as being beneficial for researchers. Additionally, none of the interventions evaluated cost-effectiveness (see also ^(15)^). Making the best use of available resources is crucial considering economic constraints of many small restaurants and takeaways. Whilst none of the included studies mentioned that any of the included restaurants and takeaways offered online food deliveries, if online deliveries are offered, this could limit the exposure to some intervention components, particularly marketing materials in-store. Given the growing size of the online food delivery sector ^(55)^, future interventions and research should consider the interaction between in-store and the growing online food delivery market.

Policymakers who want to work with small restaurants and takeaways should be mindful of potential resource constraints and adopt flexible approaches with scope for restaurants to tailor interventions to their needs. Partnering with other stakeholders such as local business associations as well as building rapport with restaurant owners can facilitate recruitment. In addition to drawing on the findings from our review that has systematically appraised the evidence base of interventions in small restaurants, policymakers who want to work with small businessesto make healthier changes should consider recommendations from existing toolkits on how to work with small, independent restaurants and takeaways ^(11, 56)^.

The majority of the interventions included in this review were conducted in areas broadly described as low-income or spanning multiple areas of deprivation. However, there was very little reporting on the impact the interventions may have had on reducing health inequalities. Most intervention elements were classed as belonging to the lower levels of the Nuffield Ladder which are seen as less intrusive and require more agency, and therefore are less likely to reduce health inequalities. In the future, researchers should consider reporting on neighbourhood levels of deprivation or collecting consumer demographic information to better assess how the interventions of interest might impact health inequities.

## Conclusion

Interventions to encourage healthy eating in small, independent, or local restaurants and hot food takeaways report mostly limited positive effects. The 13 included interventions reflect a narrow set of countries (conducted in the USA or the UK) and over the past 20 years (published between 2004 and 2020). Most interventions used less intrusive strategies (e.g. providing information, enabling choice), although we found that more intrusive interventions can be acceptable to business owners if they are inexpensive, low-effort, and not perceived as threatening customer satisfaction. Almost all interventions targeted the behaviour of both customers (e.g. menu labelling) and restaurant staff (e.g. cooking practices). However, the small number and poor quality of included studies hinders inference. More high-quality studies of interventions with objective purchase and consumption measures are needed to inform substantive policy-led actions.

## Supplementary Material

Supplementary Materials

## Figures and Tables

**Table 1:** Eligibility criteria based on Population, Intervention, Comparison, Outcome and Study design (PICOS) ^(36)^

PICOS element	Criteria
*Population*	Those selling or purchasing food in small, local, independent or community-based restaurants or takeaway outlets. There were no restrictions by country.
*Intervention*	Consumer or business-focussed interventions must aim to alter at least one of the following:
	Ordering or purchasing habits Dietary intake or dietary behaviour Availability of foods or menu options Improve the nutritional quality of individual foods, menu items or orders Interventions conducted in multiple settings or large/chained restaurants or takeaways were excluded. National-level interventions were excluded because our focus was on actions that can be taken on the local level.*
*Comparison*	No restrictions
*Outcomes*	Studies must report at least one of the following consumer or business-focused outcomes: Orders or purchases of food/beverage items Availability of food items Nutritional quality of food items (e.g. calorie content per 100g or fruit and vegetables per serving) Changes in portion sizes Measured or self-reported dietary intake
*Study design*	All primary studies (i.e. excluding reviews, comments, letters, dissertations, theses, trial registrations, protocols, conference proceedings, opinion pieces)*

* Exclusion of theses, dissertations, protocols, trial registrations, and conference abstracts as well as national-level interventions was decided after review had begun.

**Table 2:** The Nuffield intervention ladder ^(38)^, used for categorising included interventions

*Eliminate choice*	Regulate in such a way as to entirely eliminate choice, for example through compulsory isolation of patients with infectious diseases.
*Restrict choice*	Regulate in such a way as to restrict the options available to people with the aim of protecting them, for example removing unhealthy ingredients from foods, or unhealthy foods from shops or restaurants.
*Guide choice through disincentives*	Fiscal and other disincentives can be put in place to influence people not to pursue certain activities, for example through taxes on cigarettes, or by discouraging the use of cars in inner cities through charging schemes or limitations of parking spaces.
*Guide choice through incentives*	Regulations can be offered that guide choices by fiscal and other incentives, for example offering tax-breaks for the purchase of bicycles that are used as a means of travelling to work.
*Guide choice through* *changing the default* *policy*	For example, in a restaurant, instead of providing chips as a standard side dish (with healthier options available), menus could be changed to provide a more healthy option as standard (with chips as an option available).
*Enable choice*	Enable individuals to change their behaviours, for example by offering participation in a NHS ‘stop smoking’ programme, building cycle lanes, or providing free fruit in schools.
*Provide information*	Inform and education the public, for example as part of campaigns to encourage people to walk more or eat five portions of fruit and vegetables per day.
*Do nothing or simply* *monitor the current* *situation*	/

**Table 3:** Study quality assessment of included studies using MMAT ^(26)^

Study	Intervention name [or description when none given]	MMAT study design group	Q1	Q2	Q3	Q4	Q5
Ayala 2017 ^(47)^	*Kids Choice Restaurant Program (KCRP)*	2	N	Y	Y	C	Y
Bagwell 2014 ^(42)^	*London Healthier Catering Commitment*	5	Y	Y	Y	C	Y
Chen 2011 ^(49)^	*[Galerias Restaurant intervention]*	4	Y	C	C	N	Y
Fitzgerald 2004 ^(46)^	*Healthy Dining Program (HDP)*	3	C	Y	Y	C	C
Goffe 2019 ^(43)^	*[Fish and Chip Wholesaler Study]*	5	N	Y	Y	C	N
Hillier-Brown 2019 ^(39)^	*Takeaway Masterclass*	5	N	Y	Y	C	N
Lee-Kwan 2013 ^(45)^	*Baltimore Healthy Carryouts*	3	Y	Y	C	Y	Y
Lee-Kwan 2015 ^(44)^	*Baltimore Healthy Carryouts*	3	Y	Y	Y	Y	Y
Ma 2018 ^(13)^	*Healthy Chinese Take-out Initiative*	3	Y	Y	C	C	Y
McNally 2020 ^(48)^	*Fundraising - Healthy Eating Incentive*	2	C	Y	Y	C	Y
Nevarez 2013 ^(41)^	*Salud Tiene Sabor*	5	Y	Y	N	N	Y
Nothwehr 2013 ^(40)^	*[Signposting to Healthy Meals]*	5	N	Y	Y	C	N

MMAT, Mixed Methods Appraisal Tool. N, No. Y, Yes; C, Can’t tell. MMAT study design group: 2 = randomised controlled trial; 3 = quantitative non-randomised study; 4 = quantitative descriptive study; 5 = mixed methods study. Questions for each study design were as follows are in Appendix 2B.

**Table 4:** Intervention name, location, study design and stakeholders involved in included studies

Author(s)	Intervention name	Location	Study design	Stakeholders involved (other than researchers/businesses)	Business sample size
*Restaurant studies*					
Ayala 2017 ^(47)^	The Kids’ Choice Restaurant Program (KCRP)	USA, San Diego County (CA)	RCT	/	8
Chen 2011 ^(49)^	/ [Galerias Restaurant intervention]	USA, Seattle (WA)	Quantitative descriptive study	Local government/health authority; Local community organisation or non-governmental organisation (NGO)	1
Fitzgerald 2004 ^(46)^	Healthy Dining Program (HDP)	USA, suburban (no further detail provided)	Quantitative non-randomised	Local government/health authority; Other: Local advertisement agency	9
McNally 2020 ^(48)^	Fundraising-Healthy Eating Incentive	USA, San Diego County (CA)	RCT	Other: School district	1
Nevarez 2013 ^(41)^	Salud Tiene Sabor	USA, Los Angeles (CA)	Mixed methods	Local government/health authority; Local community organisation or non-governmental organisation (NGO); Other: Community health workers	7
Nothwehr 2013 ^(40)^	/ [Signposting to Healthy Meals]	USA, small towns in rural Iowa	Mixed methods	/	4
*Takeaway studies*					
Hillier- Brown 2019 ^(39)^	Takeaway Masterclass	UK, North East England	Mixed methods	Local government/health authority; Other: Industry expert	18
Lee-Kwan 2013 ^(45)^ & Lee-Kwan 2015 ^(44)^	Baltimore Healthy Carry-outs	USA, Baltimore (MD)	Quantitative non-randomised	Other: Local artist (menu design)	8
Ma 2018 ^(13)^	Healthy Chinese Take-Out Initiative	USA, Philadelphia (PA)	Quantitative non-randomised	Local government/health authority; Local community organisation or non-governmental organisation (NGO); Trade association or industry group	206 participated in intervention training, 40 measured at follow-up
*Restaurant & Takeaway studies*					
Bagwell 2014 ^(42)^	Healthier Catering Commitment	UK, 12 London boroughs	Mixed methods	Local government/health authority	77
Goffe 2019 ^(43)^	/ [Fish and Chip Wholesaler Study]	UK, northern England	Mixed methods	Trade association or industry group	12

USA, United States of America. RCT, randomised controlled trial. WA, Washington. CA, California. UK, United Kingdom. MD, Maryland. PA, Pennsylvania.

**Table 5:** Included interventions coded by the Nuffield intervention ladder ^(38)^

	Customer-level aspects of intervention	Business-level aspects of intervention
**Eliminate choice**	/	/
**Restrict choice**	/	Reducing sugar, fat, and salt content of foods, e.g. by changing cooking oil practices, products used ^(13, 39, 42)^
**Guide choice through disincentives**	/	/
**Guide choice through incentives**	Healthy meal deal options ^(39, 44, 45)^ Donation made to local school with every healthy meal purchased ^(48)^	Encouraging smaller portions (including by providing free smaller sized packaging) ^(43)^ Free first stocking of healthier snacks ^(44, 45)^ Subsidies offered to trial new healthy meal combos ^(44, 45)^ Public pledging and goal setting for changes ^(39, 43)^ Provision of free equipment, e.g. standardised measuring spoons, paper for new menus, grilling equipment ^(13, 44, 45)^ Give award to businesses for making healthier changes ^(42)^
**Guide choice through changing the default policy**	Providing salt shakers with smaller/reduced holes ^(39, 42)^ Let clients add salt ^(39)^ Restrict circulation of soy sauce packets ^(13)^ Move healthier alternatives to eye level ^(42)^	No or less salt or soy sauce added during cooking ^(13, 39, 42)^
**Enable choice**	Providing healthier options, e.g. salads, vegetables, steamed rice, reduced sugar products, tap water ^(39, 42, 44, 45, 47)^ Offering smaller portion sizes ^(39, 42)^ New healthier menu items ^(47, 49)^ Promoting/highlighting healthier options on menus ^(44–47)^ Calorie labelling on menus ^(41)^	Development of new healthier menus with support from a professional ^(47, 49)^ Cooking training, demonstration, taste-testing sessions, nutrition and health education/guidance for staff ^(13, 39, 41, 43–45, 47,49)^
**Provide information**	Marketing material promoting healthier options (e.g. posters, table signs) ^(40, 43–48)^ Point-of-purchase (POP) nutritional information ^(41, 48)^ Media campaign/newspaper articles ^(13, 40, 46)^ Staff promoting healthy eating ^(39, 42)^	Nutritional analysis of existing menu items ^(41, 44–46, 48)^

**Table 6:** Summary of intervention characteristics, outcome measures and main findings

Author(s): Intervention name	Target population & setting type	Intervention duration	Intervention components	Outcome measurement	Main findings relating to outcome of interest
**Restrict choice**					
*Bagwell 2014: Healthier Catering Commitment (HCC)* *^(42)^*	All patrons of participating restaurants and takeaways	Unclear	A series of criteria in relation to use of fats and oils, salt, sugar milk and spreads, fruit and vegetables, portion size and promotion of healthier options. To gain the award, businesses must meet eight out of 22 criteria. Four of the criteria are essential.	Survey of criteria uptake by businesses	**Business-level outcomes:** An average of 2.5 criteria-related changes had to be made for a business to secure the HCC award, with hot-food takeaway outlets having to make more changes (3.1) compared to dine-in restaurants (1.95).
*Hillier-Brown et al.* *2019:* *Takeaway* *Masterclass* ^(39)^	Customers of takeaway outlets	3 hours	3-hour training aiming to encourage healthier cooking practices and menu options, delivered to takeaway staff by public health professionals and an industry expert. Participating businesses were expected to commit to different goals.	Pre-assessment visits in-person (1-2 weeks before); For post- assessment, takeaways allocated to one of two methods: - in person visit and secret covert in-person visit (6- 8 weeks after) using a checklist to record practices - Telephone follow-up only (after 6-8 weeks); Semi-structured interviews with owners/managers in both groups	**Business-level outcomes:** At follow-up, takeaway outlets had achieved a median of 3 of the goals they had set for themselves (range of 1-7), representing 74% of all goals that were set. The goals that were reportedly achieved related to changing ingredients during cooking, changing cooking practices and offering salad and side vegetables and stocking water and healthier beverages.
**Guide choice through incentives**					
*Goffe et al. 2019:* *Fish and Chip* *Wholesaler Study* ^(43)^	Customers of fish & chip shops, both sit-in restaurants and takeaways	6 weeks	Engagement event held by the wholesaler as well as two experienced shop owners, emphasising portion control. Invitees were shop owners, managers and staff. Owners/managers unable to attend the event but interested in its content were visited by wholesaler staff.	Covert observations: availability of smaller portion meals, weight of meal components; Sales data; Customer surveys	**Business-level outcomes:** There was an increase in the number of venues offering smaller portion meals, from 6 at baseline to 8 at 6-weeks post-intervention. Reduced sizes for both regular and smaller meals (these decreases were attributable to a lower weight of chips). **Customer-level outcomes:** Smaller portion meals made up a mean of 14.2% of meals sold pre- and 21.2% of meals sold post- intervention, although the data was insufficient for proper analysis. 20% of surveyed customers reported having bought a smaller portion meal.
*Lee-Kwan et al. 2013* *& Lee-Kwan et al.* *2015:* *Baltimore Healthy* *Carry-outs* ^(44, 45)^	Customers of takeaway outlets located in urban low-income, majority African- American neighbourhood	6 months	Changing menu boards and labelling to highlight healthier options, point-of-purchase promotion (phase 1), offering and promoting (new and existing) healthy sides and beverages (phase 2) and promotion of new combination meals and altering preparation methods (phase 3).	Process evaluation data (reach, dose, fidelity): Sales receipts; site visit evaluations; intervention exposure surveys with customers (45) Sales receipts data (44)	**Customer-level outcomes:** The intervention group saw significantly increased odds of healthy entree units sold (phase 2) and healthy side and beverage units sold (phase 2 & 3) compared to baseline. The comparison group recorded increased odds of healthy side and beverage units sold (phase 1 & 3) compared to baseline. In phase 2 & 3, the intervention group recorded significantly higher odds of total healthy items sold compared to baseline, whilst odds in the comparison group were unchanged. There was a significant interaction by intervention in phases 2 & 3 for healthy sides and beverages. There were also significant increases in revenue of healthy products in the intervention group and overall revenue was significantly larger in the intervention group than control.
*Ma et al. 2018:* *Healthy Chinese Take-Out Initiative* ^(13)^	All patrons of included restaurants located in low-income neighbourhoods with high proportions of ethnic minority residents	3 years	Low-sodium cooking training and demonstrations, low-sodium recipes, mass-media campaign, and annual booster training for restaurant staff.	Sodium content mg/g of foods measured from laboratory analysis	**Business-level outcomes:** Significant and sustained reduction in the sodium content of all three target dishes in participating restaurants, although sodium content remained above USDA’s guideline intake for a single meal.
*McNally et al. 2020: Fundraising-Healthy Eating Incentive* ^(48)^	Families and children in a school district with a high share of Hispanic / Latino residents	4 days	Intervention 1: Participants received a dine out financial promotion (fundraising incentive for the school wellness programme) for the selected restaurant, with a poster promoting the menu options and nutrition information displayed at point of purchase. Participants had 15% of their total bill donated. Intervention 2: Same as intervention 1 but incentive amount was raised to an additional 10% on top of the 15% if ordering healthy items from the menu.	Sales data and receipts; customer surveys	**Customer-level outcomes:** Of the items ordered during intervention 1, 15.6% were healthy items, compared to 21.1% intervention 2. Differences between the interventions were insignificant. Healthy orders during both interventions were significantly higher compared to follow-up and higher but insignificant compared to baseline.
**Enable choice**					
*Ayala et al. 2017: The Kids’ Choice Restaurant Program (KCRP)* ^(47)^	All restaurant patrons in an area with a high share of Hispanic / Latino residents	8 weeks	Intervention 1: New menu with healthier options. Intervention 2: New menu combined with in-restaurant marketing and employee training.	Store-level weekly sales in dollars and units	**Customer-level outcomes:** Sales of new healthy children’s menu items occurred immediately and increased moderately during the intervention period, but decreased in the post-intervention period in both conditions. Sales of existing children’s menu items increased in the condition 1, but decreased in condition 2.
*Chen et al. 2011: Galerias Restaurant Intervention* ^(49)^	All restaurant patrons of a restaurant in an area with a growing Latino population, specifically targeting customers with diabetes	6 weeks	New menu insert with healthier options.	Number of items ordered from the new menu insert; customer survey	**Customer-level outcomes:** 11.6% of dishes sold were from the new lighter menu with 90% of patrons open to choosing healthier items.
*Fitzgerald et al. 2004:* *Healthy Dining Program (HDP)* ^(46)^	All restaurant customers	8 weeks	Identification and labelling of heart-healthy menu items, combined with promotional campaign.	Restaurant sales log sheets	**Customer-level outcomes:** Small increase in the proportion of heart-healthy menu sales during the 8-week campaign, from 30% before, to 32% after, although this was not statistically significant and there was great heterogeneity between restaurants.
*Nevarez et al. 2013:* *Salud Tiene Sabor* ^(41)^	All patrons of selected restaurants in a low-income, majority Latino community	10 months - 1 year	Calorie labelling of menus and additional nutrition information brochures available at point of sale. Restaurants also received cooking advice from a dietitian on how to modify their menu items to be healthier.	Calorie content, description and variety of foods collected via the Food and Beverage Environmental Assessment tool; Customer interviews; Interviews with restaurant owners	**Business-level outcomes:** Nearly half of all entrees (42%) and side dishes (41%) sold met the Los Angeles County Worksite standards on calories per serving. **Customer-level outcomes** Nearly half of all patrons (46%) who said they had noticed the calorie information reporting that the calorie information influenced their purchasing decision. Generally, about one third of patrons stated that their point-of-purchase decision was influenced by the calorie information.
**Provide information**					
*Nothwehr et al. 2013:* *Signposting to Health* *Meals* ^(40)^	Customers of participating restaurants, predominantly White (98%) population	1 year	Plastic signs were positioned on tables that outlined strategies to make healthier orders. An entryway or front window sign also highlighted the healthy options. Local newspapers reported on the initiative.	Self-administered customer surveys; Interviews with owners; Order slips	**Customer-level outcomes:** Around 34% of customers surveyed who saw the signs reported that it influenced what they ordered. There was no significant time trend of healthy ordering behaviour.
